# Author Correction: RNaseH2A downregulation drives inflammatory gene expression via genomic DNA fragmentation in senescent and cancer cells

**DOI:** 10.1038/s42003-025-09392-y

**Published:** 2025-12-30

**Authors:** Sho Sugawara, Ryo Okada, Tze Mun Loo, Hisamichi Tanaka, Kenichi Miyata, Masatomo Chiba, Hiroko Kawasaki, Kaoru Katoh, Shizuo Kaji, Yoshiro Maezawa, Koutaro Yokote, Mizuho Nakayama, Masanobu Oshima, Koji Nagao, Chikashi Obuse, Satoshi Nagayama, Keiyo Takubo, Akira Nakanishi, Masato T. Kanemaki, Eiji Hara, Akiko Takahashi

**Affiliations:** 1 Division of Cellular Senescence, Cancer Institute, Japanese Foundation for Cancer Research, Koto-ku, Tokyo 135-8550 Japan; 2 Department of Molecular Genetics, Medical Research Institute, Tokyo Medical and Dental University (TMDU), Bunkyo-ku, Tokyo 113-8510 Japan; 3 Biomedical Research Institute, National Institute of Advanced Industrial Science and Technology (AIST), Tsukuba, Ibaraki 305-8560 Japan; 4 Institute of Mathematics for Industry, Kyushu University, Nishi-ku, Fukuoka 819-0395 Japan; 5 Department of Endocrinology, Hematology and Gerontology, Graduate School of Medicine, Chiba University, Chiba, Chiba, 260-0856 Japan; 6 Division of Genetics, Cancer Research Institute, Kanazawa University, Kanazawa, 920-1192 Japan; 7 Laboratory of Genome Structure and Function, Graduated School of Science, Osaka University, Toyonaka, Osaka 560-0043 Japan; 8 Department of Gastroenterological Surgery, Cancer Institute Hospital, Japanese Foundation for Cancer Research, 135-8550 Tokyo, Japan; 9 Department of Surgery, Uji-Tokushukai Medical Center, Kyoto, 611-0041 Japan; 10 Department of Stem Cell Biology, Research Institute, National Center for Global Health and Medicine, Tokyo, 162-8655 Japan; 11 Department of Chromosome Science, National Institute of Genetics, Research Organization of Information and Systems (ROIS), Yata 1111, Mishima, Shizuoka, 411-8540 Japan; 12 Department of Genetics, The Graduate University for Advanced Studies (SOKENDAI), Yata 1111, Mishima, Shizuoka, 411-8540 Japan; 13 Department of Molecular Microbiology, Research Institute for Microbial Diseases, Osaka University, Suita, Osaka, 565-0871 Japan; 14 Advanced Research & Development Programs for Medical Innovation (PRIME), Japan Agency for Medical Research and Development (AMED), Chiyoda-ku, Tokyo 104-0004 Japan; 15 Cancer Cell Communication Project, NEXT-Ganken Program, Japanese Foundation for Cancer Research, Tokyo, 135-8550 Japan

**Keywords:** Senescence, Tumour-suppressor proteins

Correction to: *Communications Biology* 10.1038/s42003-022-04369-7, published online 28 December 2022

In the version of the article initially published, due to a figure preparation error, in Fig. 5d, the plot for CXCL10 was an inadvertent duplicate of the adjacent IFNB1 plot. The figure is now amended in the HTML and PDF versions of the article.

