# Supplementary material for: RNaseH2A downregulation drives inflammatory gene expression via genomic DNA fragmentation in senescent and cancer cells
Source: Commun Biol. 2022 Dec 28;5:1420. doi: 10.1038/s42003-022-04369-7 (PMC9797495; doi:10.1038/s42003-022-04369-7)
Supplement: Supplementary file 2 — Supplementary information [file 42003_2022_4369_MOESM2_ESM.pdf]

Supplementary information for

RNaseH2A downregulation drives inflammatory gene expression via genomic DNA fragmentation in senescent and cancer cells

Sho Sugawara, Ryo Okada, Tze Mun Loo, Hisamichi Tanaka, Kenichi Miyata, Masatomo Chiba, Hiroko Kawasaki, Kaoru Katoh, Shizuo Kaji, Yoshiro Maezawa, Koutaro Yokote, Mizuho Nakayama, Masanobu Oshima, Koji Nagao, Chikashi Obuse, Satoshi Nagayama, Keiyo Takubo, Akira Nakanishi, Masato T. Kanemaki, Eiji Hara, Akiko Takahashi*

* Corresponding author.

E-mail: akiko.takahashi@jfcr.or.jp

This supplementary information file includes:

Supplementary Figure 1 to 8

Uncropped original immunoblots and gel images

Supplementary Data 1 to 5 legend

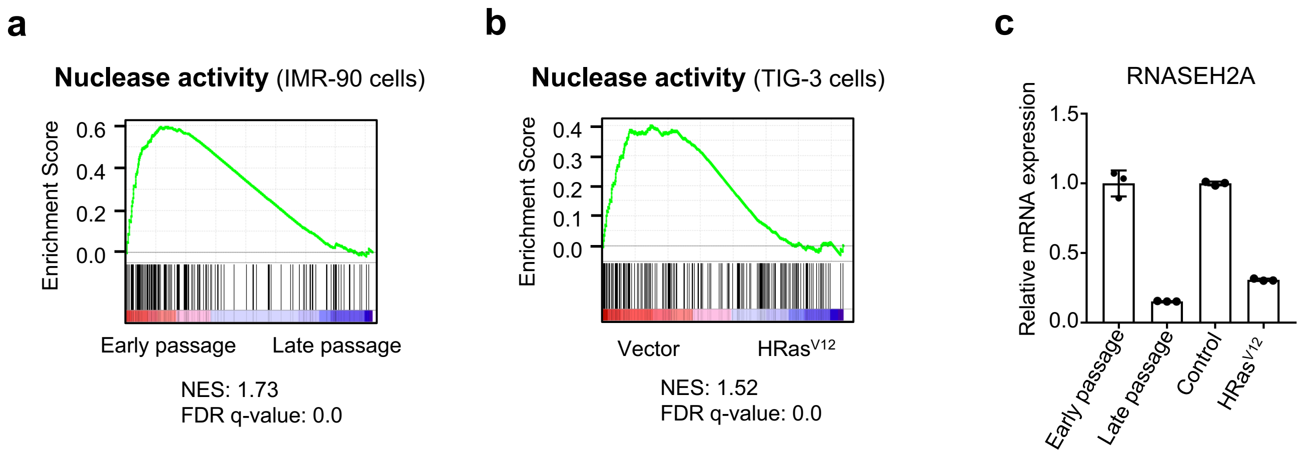

Supplementary Fig. 1

a Gene set enrichment analysis (GSEA) using the gene set for the nuclease activity of pre-senescent IMR-90 (early passage) and senescent IMR-90 cells (late passage). NES: normalised enrichment score. FDR: false discovery rate. **b** GSEA using the gene set for the nuclease activity of TIG-3 cells infected with retrovirus encoding oncogenic Ras (HRas^{V12}) or empty vector. **c** Quantitative reverse transcription PCR analysis of ribonuclease H2 subunit A gene expression in early-passage, late-passage, control or HRas^{V12}-induced senescent IMR-90 cells. Error bars indicate the mean ± SD of technical triplicates.

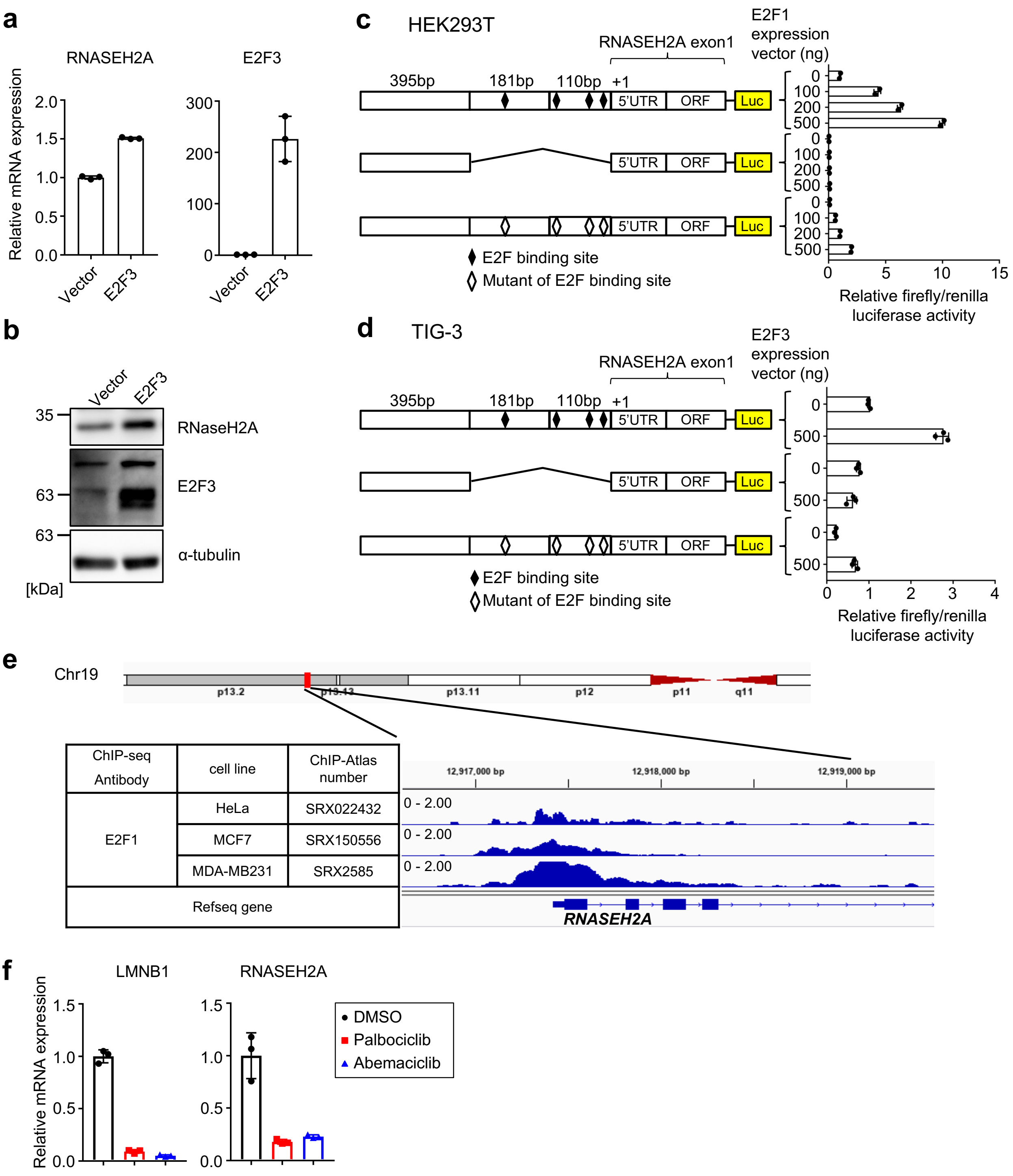
a
RNASEH2A
E2F3
Relative mRNA expression
Vector
E2F3
b
Vector
E2F3
RNaseH2A
E2F3
α-tubulin
[kDa]
c
HEK293T
RNASEH2A exon1
E2F1 expression vector (ng)
395bp
181bp
110bp
+1
5'UTR
ORF
Luc
E2F binding site
Mutant of E2F binding site
Relative firefly/renilla luciferase activity
d
TIG-3
E2F3 expression vector (ng)
e
Chr19
ChIP-seq Antibody
cell line
ChIP-Atlas number
E2F1
HeLa
SRX022432
MCF7
SRX150556
MDA-MB231
SRX2585
Refseq gene
RNASEH2A
f
LMNB1
RNASEH2A
DMSO
Palbociclib
Abemaciclib

Supplementary Fig. 2

a TIG-3 cells were transfected with the E2F3 expression vector. After 48 h, cells were subjected to quantitative reverse transcription PCR (RT-qPCR) against the indicated genes. Error bars indicate the mean ± SD of technical triplicates. **b**. Western blot of TIG-3 cells as in **a** using the antibodies indicated on the right. Alpha-tubulin was used as a loading control. **c** Left: Schematic representation of the reporter construct of the human ribonuclease H2 subunit A (*RNASEH2A*) gene promoter used in the analysis. Firefly luciferase is presented as Luc. The E2F-binding element or its mutant is presented as a black or white rhombus. Right: HEK293T cells were transfected with the E2F1 expression vector, the indicated reporter plasmids, and a *Renilla* luciferase plasmid as an internal control. After 48 h of transfection, luciferase activities were measured. Error bars indicate the mean ± SD of technical duplicates. **d** Left: Schematic representation of the reporter construct of human *RNASEH2A* gene promoter used in the analysis. Right: TIG-3 cells were transfected with the E2F3 expression vector, the indicated reporter plasmids, and a *Renilla* luciferase plasmid as an internal control. After 48 h of transfection, luciferase activities were measured. Error bars indicate the mean ± SD of technical triplicates. **e** Chromatin immunoprecipitation (ChIP) sequencing data of E2F1 in the RNASEH2A gene locus of Chr19 was extracted from the ChIP-Atlas database. On the left side, the target molecule (E2F1), cell type (HeLa, MCF7, or MDA-MB231), and ChIP-Atlas number are described. **f** RT-qPCR of the indicated gene expression in TIG-3 cells treated with 1 μM cyclin-dependent kinase 4/6 inhibitors for 7 days. Error bars indicate the mean ± SD of technical triplicates.

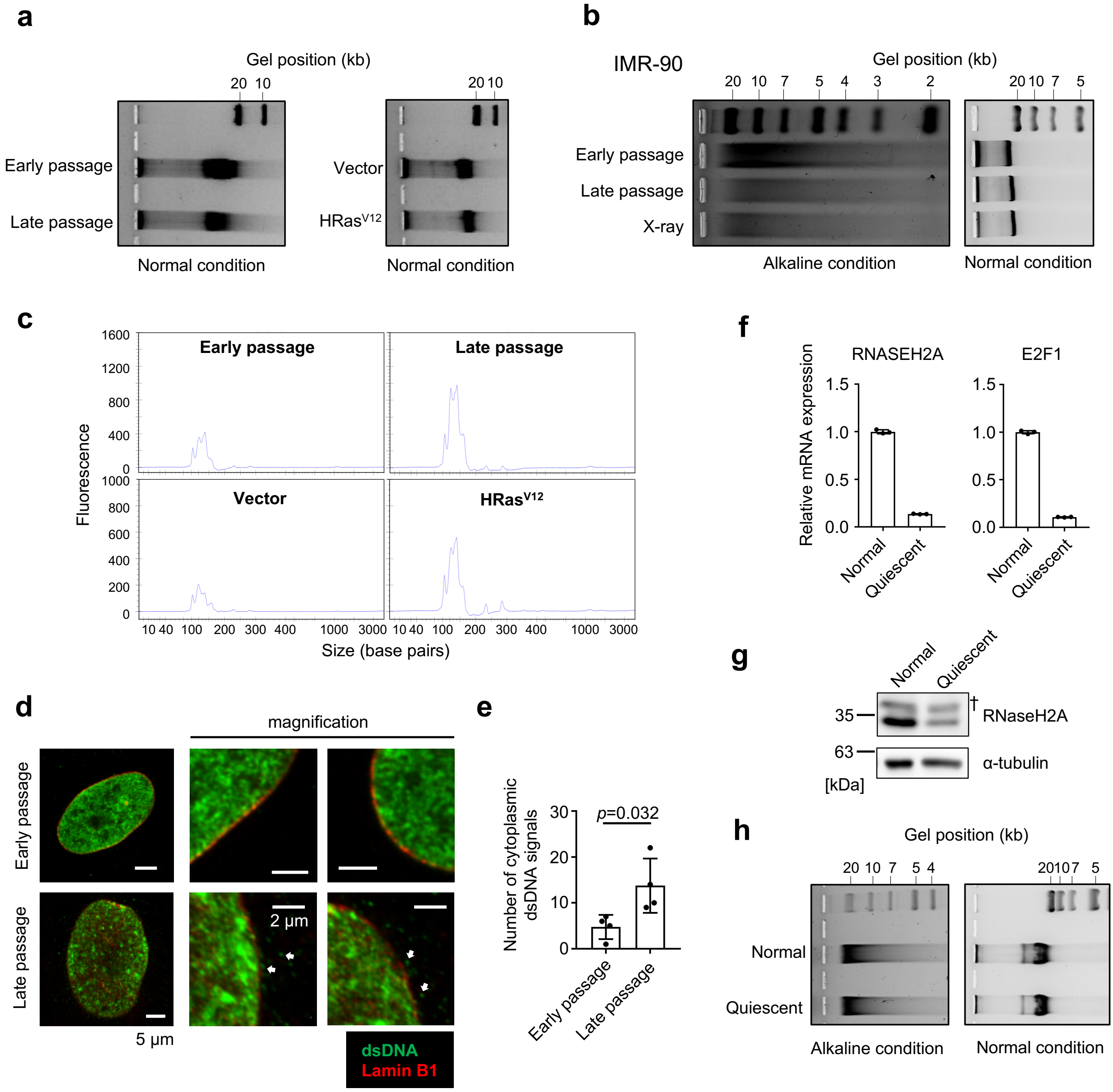

a
Gel position (kb)
20 10
Early passage
Late passage
Normal condition
Vector
HRasV12
Normal condition
b
IMR-90
Gel position (kb)
20 10 7 5 4 3 2
Early passage
Late passage
X-ray
Alkaline condition
Normal condition
c
Early passage
Late passage
Vector
HRasV12
Fluorescence
Size (base pairs)
d
magnification
Early passage
Late passage
5 μm
2 μm
dsDNA
Lamin B1
e
Number of cytoplasmic dsDNA signals
p=0.032
Early passage
Late passage
f
RNASEH2A
E2F1
Relative mRNA expression
Normal
Quiescent
g
Normal
Quiescent
35
63
[kDa]
RNaseH2A
α-tubulin
h
Gel position (kb)
Normal
Quiescent
Alkaline condition
Normal condition

Supplementary Fig. 3

a Genomic DNA was extracted from pre-senescent (early passage, vector) and senescent (late passage, oncogenic Ras [$HRas^{V12}$]) TIG-3 cells and subjected to gel electrophoresis without alkaline hydrolysis (normal condition). **b** Genomic DNA was extracted from pre-senescent (early passage) and senescent (late passage, X-ray) IMR-90 cells and subjected to gel electrophoresis with or without alkaline hydrolysis. **c** Pre-senescent TIG-3 cells were rendered senescent by serial passage or ectopic induction of $HRas^{V12}$, and the cytoplasmic fraction was isolated followed by fragment length analysis with a LabChip GX Touch Nucleic Acid Analyzer (Perkin Elmer). **d** Pre-senescent TIG-3 cells were rendered senescent by serial passage (late passage) and then subjected to immunofluorescence staining for double-stranded DNA (dsDNA; green) and Lamin B1 (red) using a super-resolution microscope. Scale bars denote 5 (left panel) or 2 μm (other panels). White arrows denote dsDNA fragments in cytoplasm. **e** The numbers of cytoplasmic dsDNA signals were counted as in **c**. Error bars indicate the mean ± SD of biological replicates. Two-tailed Student's *t*-test. **f** TIG-3 cells were rendered quiescent by serum starvation and subjected to quantitative reverse transcription PCR for indicated genes. Error bars indicate the mean ± SD of technical triplicates. **g** Western blot of TIG-3 cells as in **e** using antibodies shown at right. Alpha-tubulin was used as a loading control. †: Non-specific signal. **h** Genomic DNA was extracted from TIG-3 cells as in **e** and subjected to gel electrophoresis with or without alkaline hydrolysis. All data are representative of at least three biological replicates.

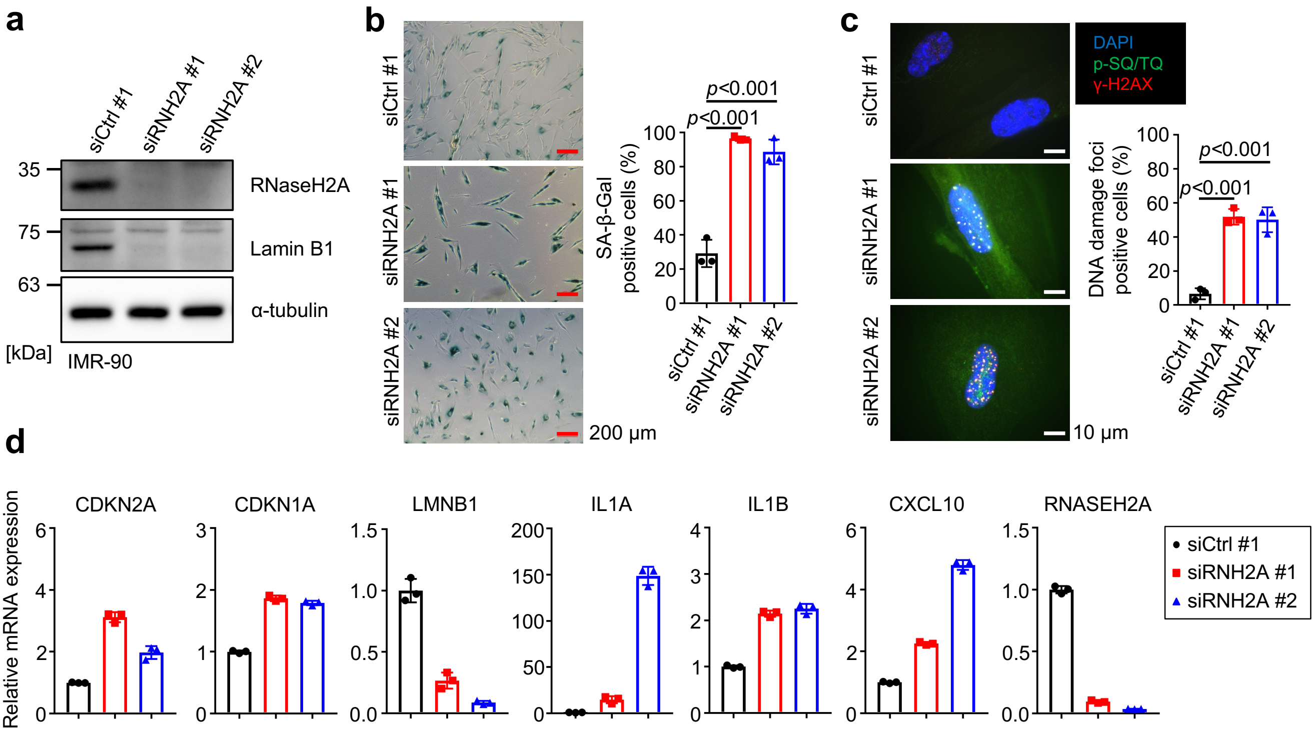

Supplementary Fig. 4

a Western blot of pre-senescent IMR-90 cells treated with the indicated small interfering RNAs (siCtrl#1, RNH2A#1 and #2) three times for 72h each. Alpha-tubulin was used as a loading control. **b** Left: Representative images of SA-β-Gal staining of IMR-90 cells as in **a**. Scale bars indicate 200 µm. Right: Quantification of SA-β-Gal positive cells. The graphs indicate the percentage of SA-β-Gal positive cells. Error bars indicate the mean ± SD of biological triplicates. One-way ANOVA coupled with Dunnett's multiple comparisons test. **c** Left: Representative immunofluorescence images of DNA damage marker in IMR-90 cells as in **a**. γ-H2AX (red), phospho-Ser/Thr ATM/ATR (p-SQ/TQ) substrate (green), and DAPI (blue). Scale bars indicate 10 µm. Right: Quantification of DNA damage-positive cells. The graphs indicate the percentage of nuclei containing more than two foci positive for both γ-H2AX and p-SQ/TQ. Error bars indicate the mean ± SD of biological triplicates. One-way ANOVA coupled with Dunnett's multiple comparisons test. **d** Quantitative reverse transcription PCR of the indicated genes using RNA extracted from IMR-90 cells as in **a**. Error bars indicate the mean ± SD of technical triplicates. All data are representative of at least three biological replicates.

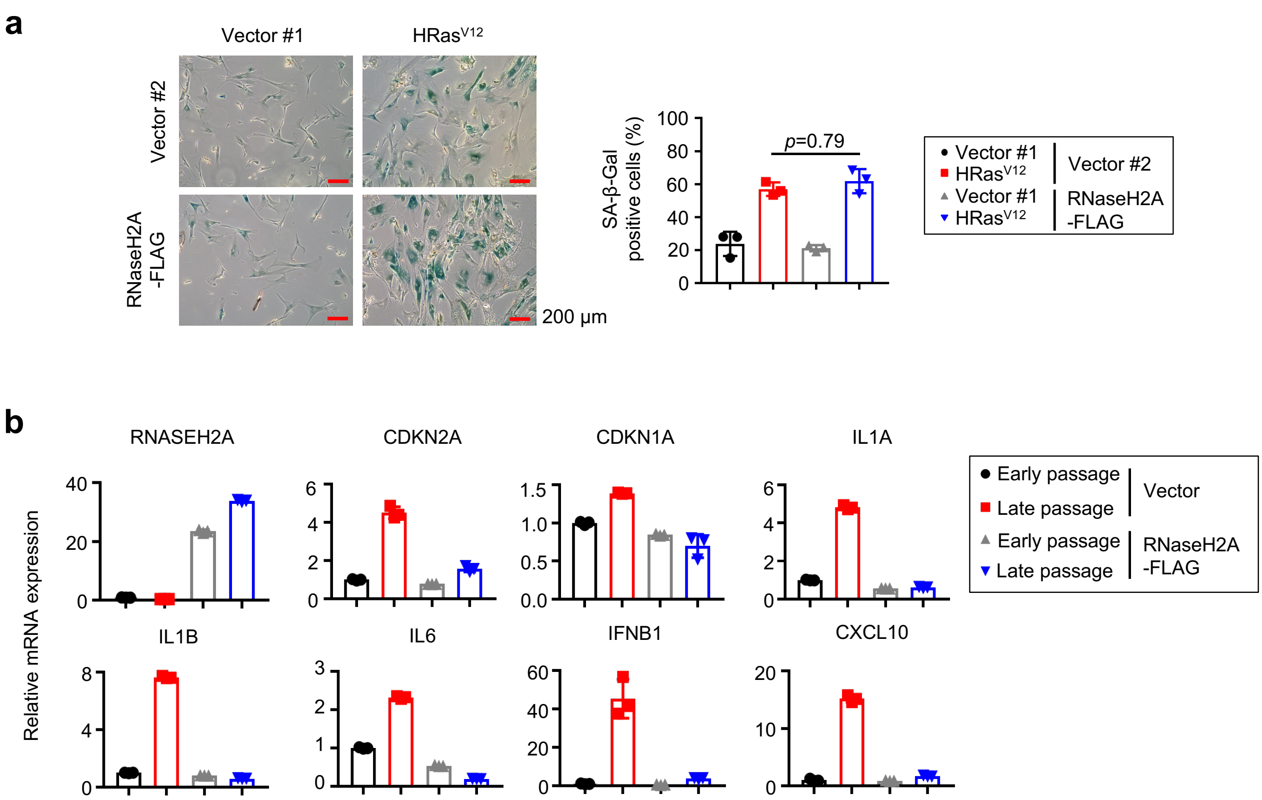

Supplementary Fig. 5

a Left: Representative images of SA-β-Gal staining of TIG-3 cells expressing oncogenic Ras (HRas^{V12}) and/or FLAG-tagged ribonuclease H2 subunit A (RNaseH2A) as in Figure 5. Scale bars indicate 200 μm. Right: Quantification of SA-β-Gal positive cells. The graphs indicate the percentage of SA-β-Gal positive cells. Error bars indicate the mean ± SD of biological triplicates. Two-way ANOVA coupled with Tukey's multiple comparisons test. **b** TIG-3 cells infected with retrovirus encoding FLAG-tagged RNaseH2A or empty vector. After puromycin selection, cells were rendered senescent by serial passage and subjected to quantitative reverse transcription PCR of the indicated genes. Error bars indicate the mean ± SD of technical triplicates. All data are representative of at least three biological replicates.

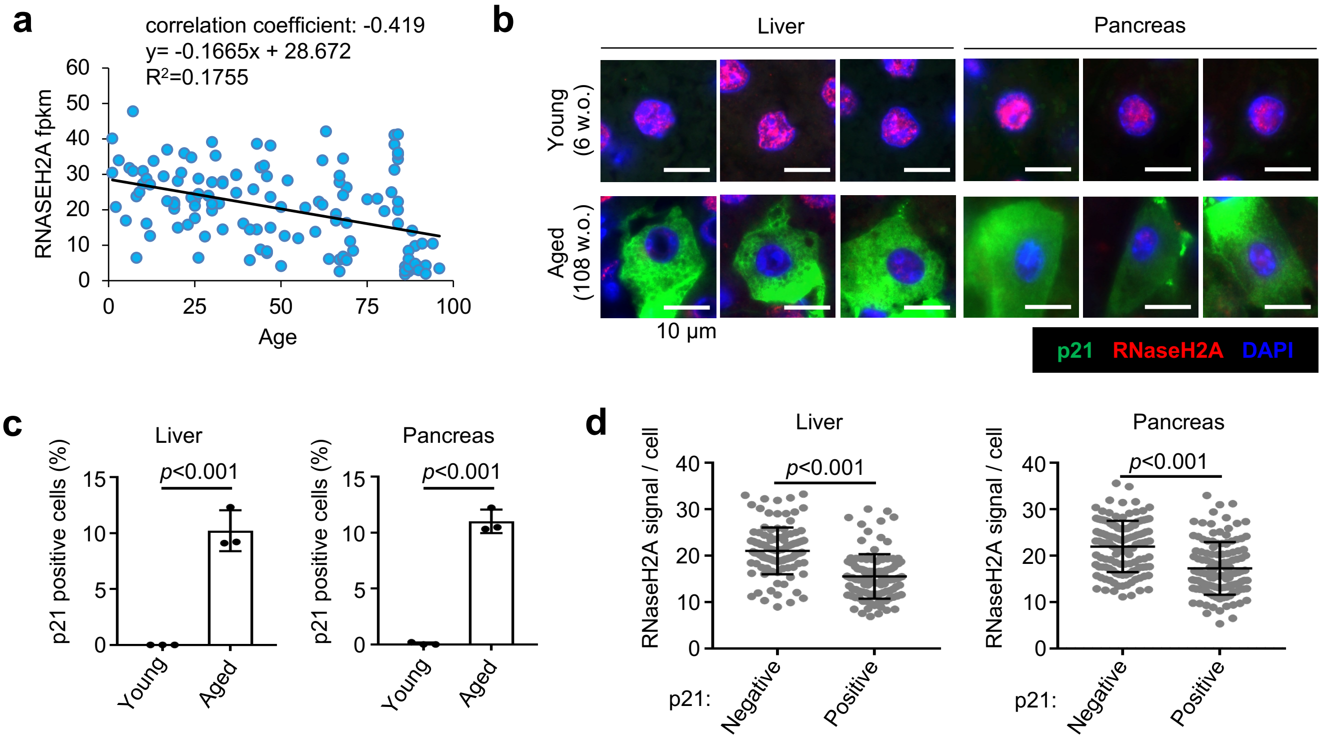

Supplementary Fig. 6

a Correlation between ribonuclease H2 subunit A (RNASEH2A) expression and age. RNA sequencing dataset of fibroblast lines derived from 130 healthy individuals ranging in age from 1 to 96 years (GSE113957). **b** Biopsy samples of young (6 weeks old) and aged (108 weeks old) C57BL/6J mice liver (left) and pancreas tissue (right) were subjected to fluorescent immunohistochemistry using antibodies against p21$^{WAF1/CIP1}$ and RNaseH2A. Scale bars indicate 10 μm. **c** Percentage of p21-positive cells as in **b** from three biological replicates. Each dot represents the percentage of p21-positive cells from at least 100 cells per experiment. Error bars indicate the mean ± SD of biological replicates. Two-tailed Student's *t*-test. **d** Quantification of the RNaseH2A signal intensity in p21-negative or p21-positive cells from aged mouse hepatic or pancreatic tissues as in **b**. Each dot represents the value of a single cell (n > 100). Error bars indicate the mean ± SD of biological replicates. Two-tailed Student's *t*-test. All data are representative of at least three biological replicates.

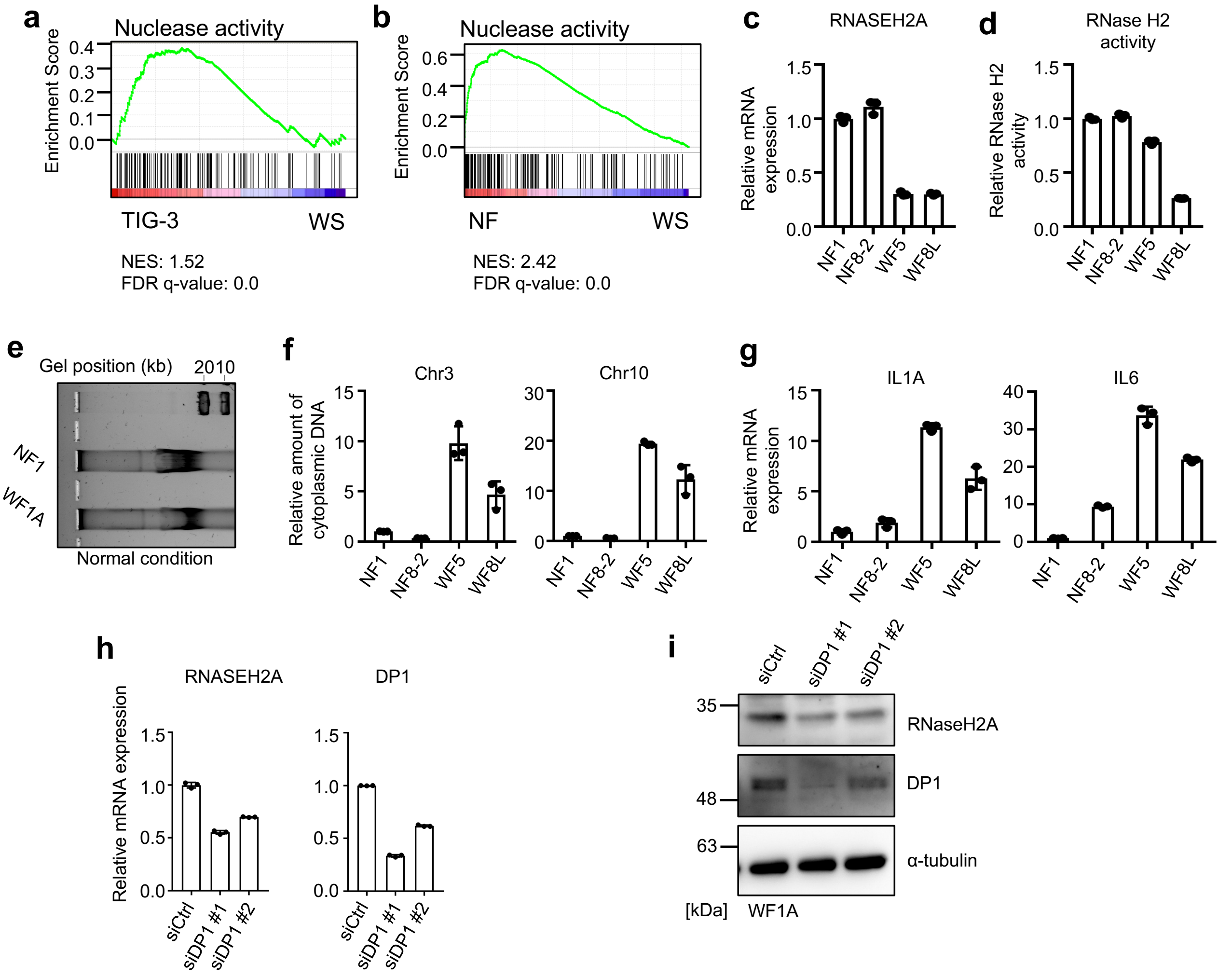

Supplementary Fig. 7

a GSEA using a gene set for nuclease activity. Expression dataset of fibroblasts (TIG-3 cells) versus fibroblasts derived from patients with Werner syndrome (WS: GSE62114). **b** GSEA using a gene set for nuclease activity. Expression dataset of normal fibroblasts (NF) versus fibroblasts derived from patients with WS (WSF: GSE48761). **c** RT-qPCR analysis of ribonuclease H2 subunit A (*RNASEH2A*) gene expression in NF1, NF8-2, WF5, and WF8L cells (15–20 population doublings). NF1, NF8: healthy human fibroblasts; WF5, WF8L: fibroblasts derived from patients with WS. Error bars indicate the mean ± SD of technical triplicates. **d** In vitro RNase H2-specific activity assay of four human fibroblast lines as in **c**. Error bars indicate the mean ± SD of technical triplicates. **e** Genomic DNA was extracted from NF1 and WF1A cells and then subjected to gel electrophoresis without alkaline hydrolysis (normal condition). A representative gel image from three biological replicates is presented. **f** Quantitative PCR analysis of chromosomal DNA in the cytoplasm of four human fibroblast lines as in **c** using primers against chromosomes 3 and 10. Error bars indicate the mean ± SD of technical triplicates. **g** RT-qPCR of the indicated genes was conducted using RNA extracted from four human fibroblast lines as in **c**. Error bars indicate the mean ± SD of technical triplicates. **h** RT-qPCR of RNASEH2A and DP1 mRNA expression in small interfering RNA-treated WF1A cells. Error bars indicate the mean ± SD of technical triplicates. **i** Western blot of WF1A cells as in **h** using the antibodies presented on the right. Alpha-tubulin was used as a loading control. All data are representative of at least three biological replicates.

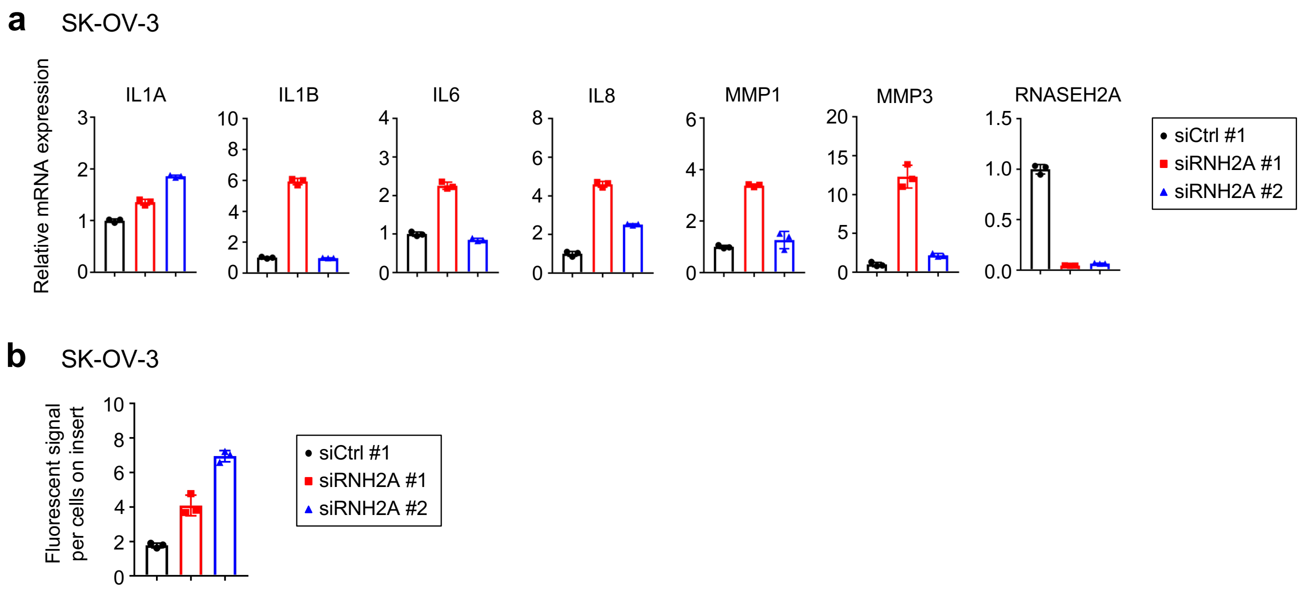

Supplementary Fig. 8

a Quantitative reverse transcription PCR of the indicated genes using RNA extracted from SK-OV-3 cells treated with the indicated small interfering RNAs. Error bars indicate the mean ± SD of technical triplicates. **b** Cell invasion assay using SK-OV-3 cells as in **a**. Error bars indicate the mean ± SD of technical triplicates. All data are representative of at least three biological replicates.

Original immunoblots in Figure 1e

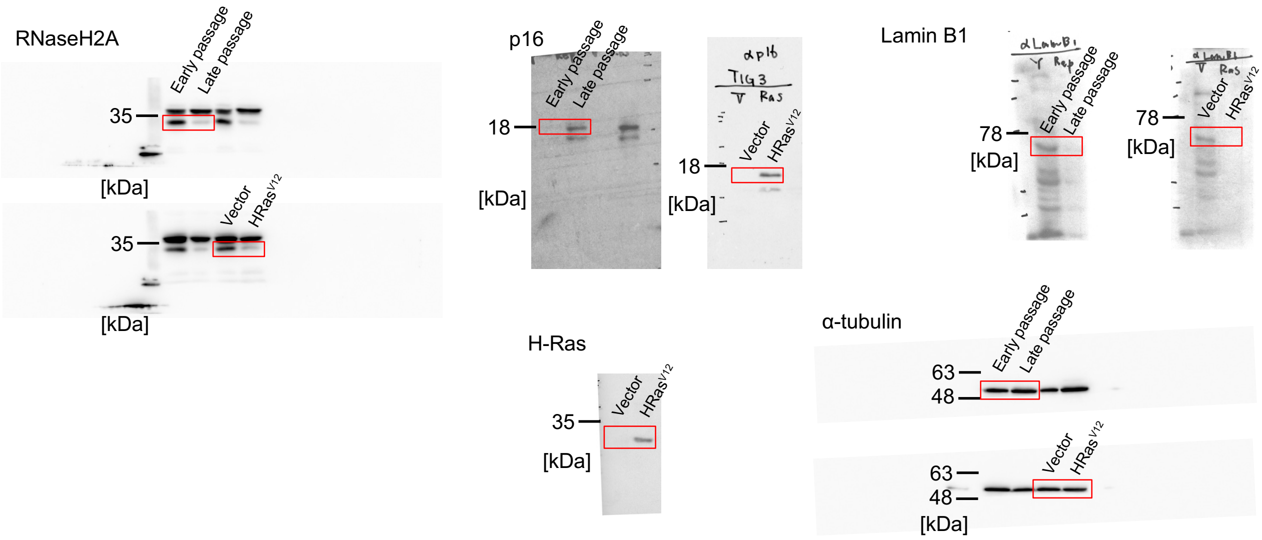

Original immunoblots in Figure 2d

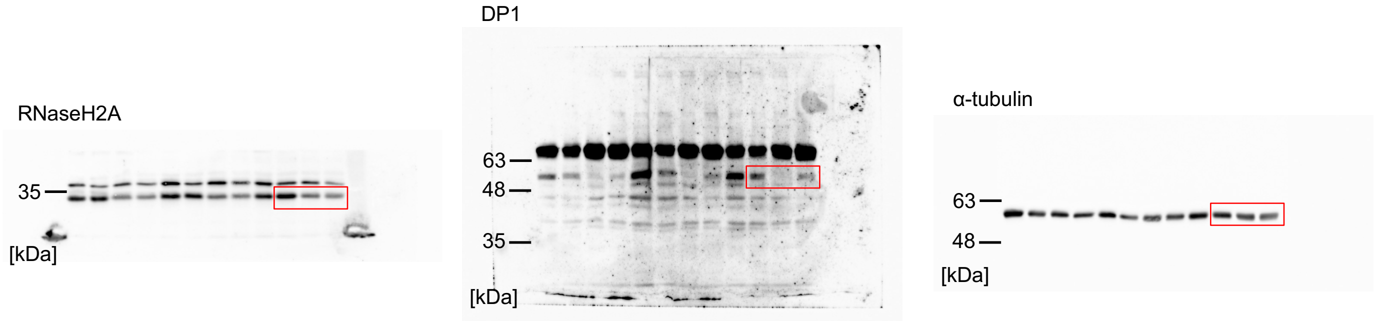

Original gel image in Figure 3a

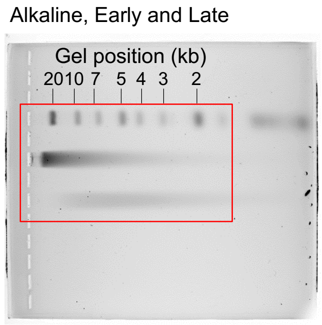

Original gel image in Figure 3b

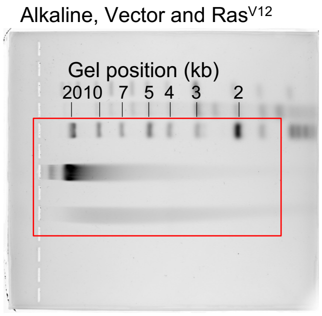

Original immunoblots in Figure 4a

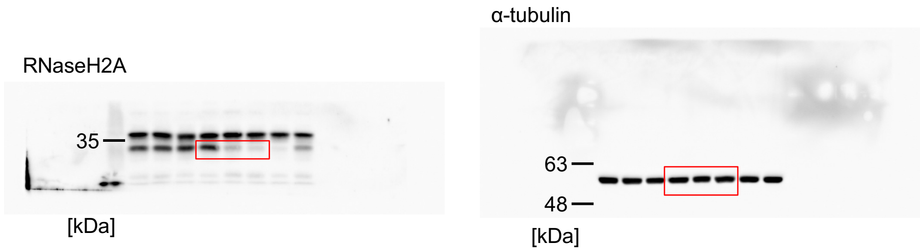

Original immunoblots in Figure 4f

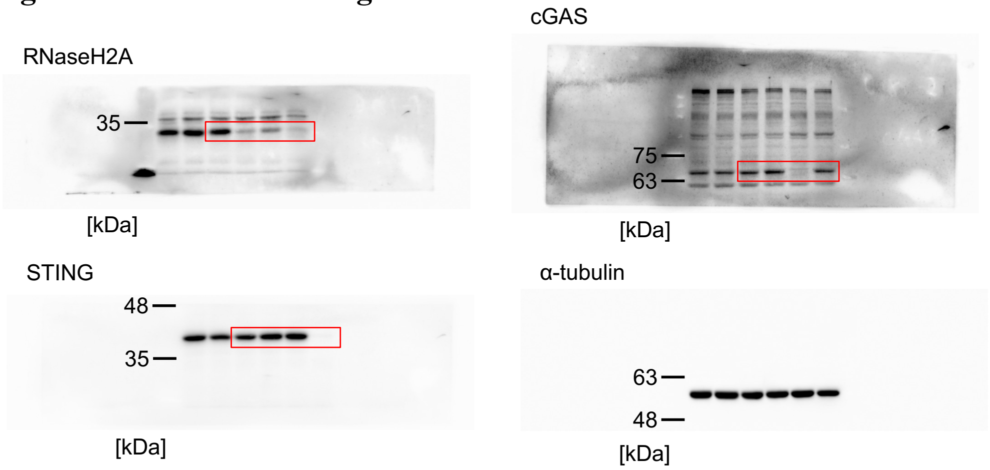

Original gel image in Figure 5a

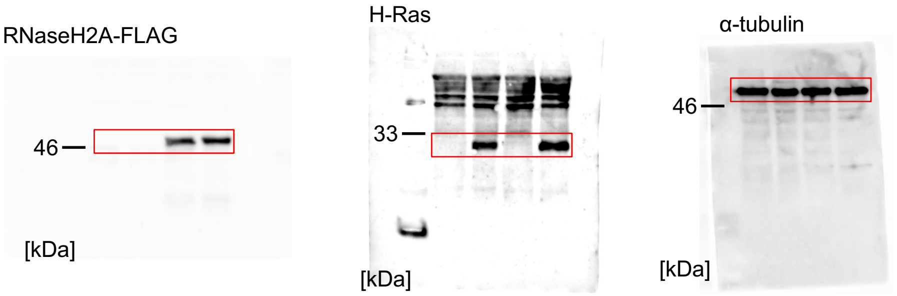

Original immunoblots in Figure 6b

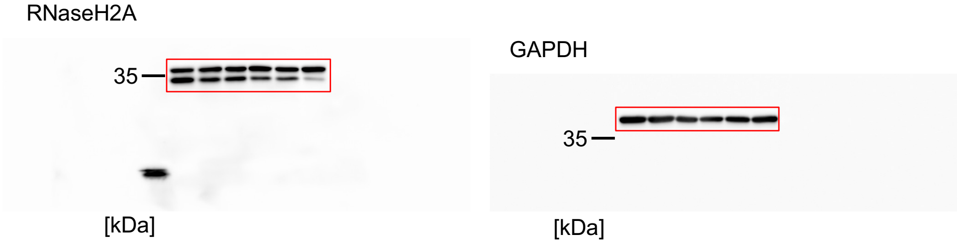

Original gel image in Figure 6d

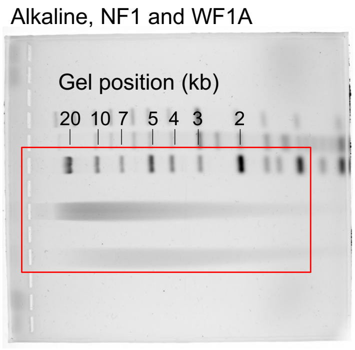

Original immunoblots in Figure 7b

RNaseH2A-mAID

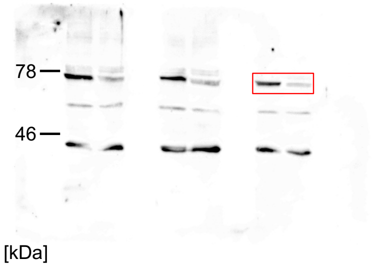

α-tubulin

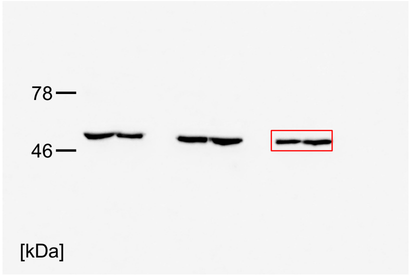

Original immunoblots in Supplementary Figure 2b

RNaseH2A

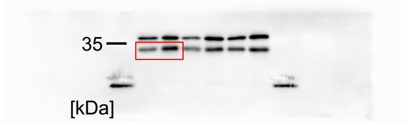

E2F3

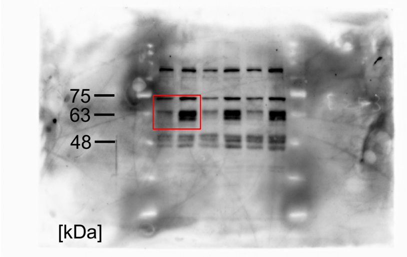

α-tubulin

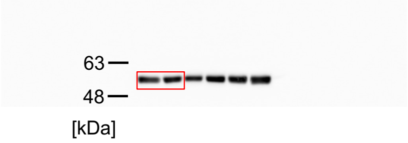

Original gel images in Supplementary Figure 3a

Normal, Early and Late

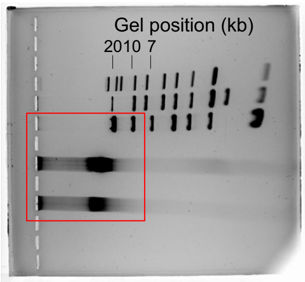

Normal, Vector and Ras^{V12}

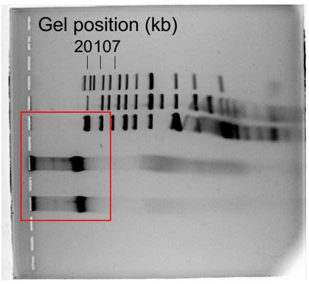

Original gel images in Supplementary Figure 3b

Alkaline

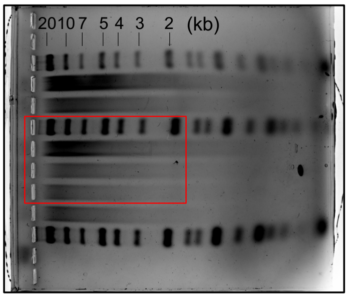

Normal

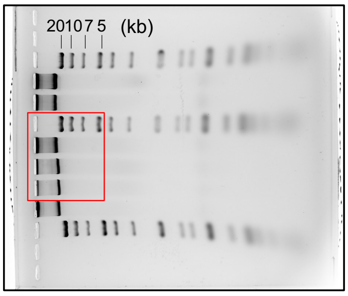

Original immunoblots in Supplementary Figure 3g

RNaseH2A

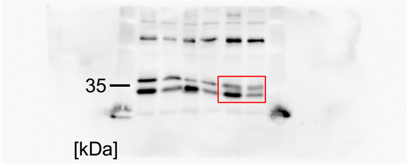

α-tubulin

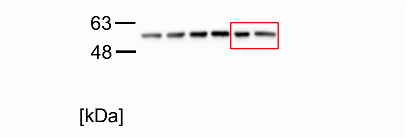

Original gel images in Supplementary Figure 3h

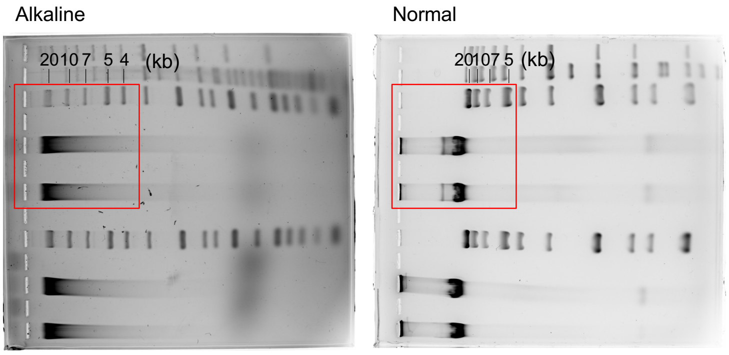

Original immunoblots in Supplementary Figure 4a

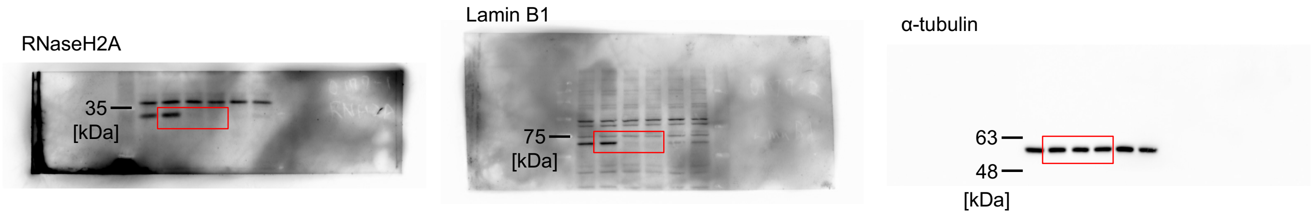

Original gel images in Supplementary Figure 7e

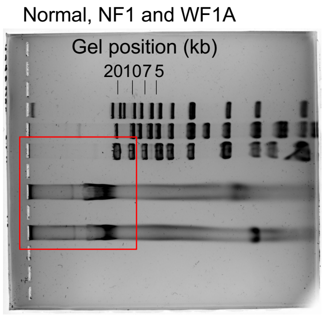

Original immunoblots in Supplementary Figure 7i

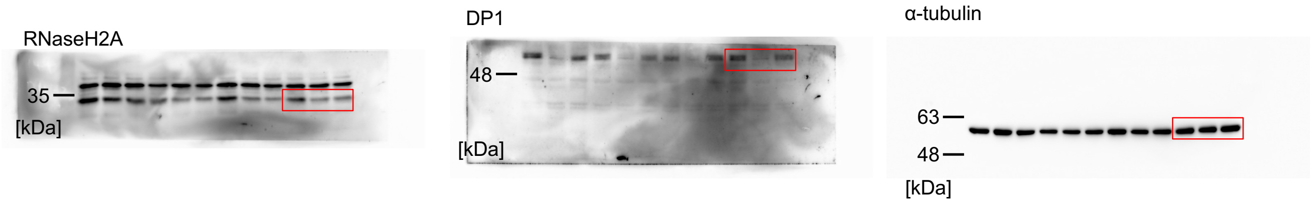

Supplementary Data 1:
Early_Late_RNA-sequence_analysis-data

Supplementary Data 2:
Vector_HRasV12_RNA-sequence_analysis-data

Supplementary Data 3:
Nuclease_activity_Gene_Set_Enrichment_Analysis_data

Supplementary Data 4:
Quantitative real-time PCR primers list.

Supplementary Data 5:
The source data behind the graphs in the paper.
