# Supplementary material for: RNaseH2A downregulation drives inflammatory gene expression via genomic DNA fragmentation in senescent and cancer cells
Source: Commun Biol. 2022 Dec 28;5:1420. doi: 10.1038/s42003-022-04369-7 (PMC9797495; doi:10.1038/s42003-022-04369-7)
Supplement: Supplementary file 3 — Description of Additional Supplementary Data [file 42003_2022_4369_MOESM3_ESM.docx]

**Description of Additional Supplementary Files**

**File name:** Supplementary Data 1

**Description:** RNA-seq data comparing early passage with late passage.

**File name:** Supplementary Data 2

**Description:** RNA-seq data comparing vector- with HRasV12-expressing cells.

**File name:** Supplementary Data 3

**Description:** GSEA data for genes contained in Nuclease activity.

**File name:** Supplementary Data 4

**Description:** Quantitative real-time PCR primers list.

**File name:** Supplementary Data 5

**Description:** The source data behind the graphs in the paper.
