# Supplementary material for: RNaseH2A downregulation drives inflammatory gene expression via genomic DNA fragmentation in senescent and cancer cells
Source: Commun Biol. 2022 Dec 28;5:1420. doi: 10.1038/s42003-022-04369-7 (PMC9797495; doi:10.1038/s42003-022-04369-7)
Supplement: Supplementary file 9 — Reporting-summary [file 42003_2022_4369_MOESM9_ESM.pdf]

nature portfolio

Corresponding author(s): Akiko Takahashi

Last updated by author(s): Nov 30, 2022

Reporting Summary

Nature Portfolio wishes to improve the reproducibility of the work that we publish. This form provides structure for consistency and transparency in reporting. For further information on Nature Portfolio policies, see our Editorial Policies and the Editorial Policy Checklist.

Statistics

For all statistical analyses, confirm that the following items are present in the figure legend, table legend, main text, or Methods section.

n/a	Confirmed	
☐	☒	The exact sample size (*n*) for each experimental group/condition, given as a discrete number and unit of measurement
☐	☒	A statement on whether measurements were taken from distinct samples or whether the same sample was measured repeatedly
☐	☒	The statistical test(s) used AND whether they are one- or two-sided<br>*Only common tests should be described solely by name; describe more complex techniques in the Methods section.*
☐	☒	A description of all covariates tested
☐	☒	A description of any assumptions or corrections, such as tests of normality and adjustment for multiple comparisons
☐	☒	A full description of the statistical parameters including central tendency (e.g. means) or other basic estimates (e.g. regression coefficient) AND variation (e.g. standard deviation) or associated estimates of uncertainty (e.g. confidence intervals)
☐	☒	For null hypothesis testing, the test statistic (e.g. *F*, *t*, *r*) with confidence intervals, effect sizes, degrees of freedom and *P* value noted<br>*Give P values as exact values whenever suitable.*
☒	☐	For Bayesian analysis, information on the choice of priors and Markov chain Monte Carlo settings
☒	☐	For hierarchical and complex designs, identification of the appropriate level for tests and full reporting of outcomes
☐	☒	Estimates of effect sizes (e.g. Cohen's *d*, Pearson's *r*), indicating how they were calculated

Our web collection on statistics for biologists contains articles on many of the points above.

Software and code

Policy information about availability of computer code

Data collection	All software is commercially or freely available and described in manuscript.
Data analysis	All software is commercially or freely available and described in manuscript. The data were analysed using Graphpad Prism v7.04.

For manuscripts utilizing custom algorithms or software that are central to the research but not yet described in published literature, software must be made available to editors and reviewers. We strongly encourage code deposition in a community repository (e.g. GitHub). See the Nature Portfolio guidelines for submitting code & software for further information.

Data

Policy information about availability of data

All manuscripts must include a data availability statement. This statement should provide the following information, where applicable:

- Accession codes, unique identifiers, or web links for publicly available datasets
- A description of any restrictions on data availability
- For clinical datasets or third party data, please ensure that the statement adheres to our policy

Nucleotide sequence data reported are available in the DDBJ Sequenced Read Archive under the accession numbers DRA009786,. The data that support the findings of this study are available in the supplementary material of this article.

Field-specific reporting

Please select the one below that is the best fit for your research. If you are not sure, read the appropriate sections before making your selection.

☒ Life sciences ☐ Behavioural & social sciences ☐ Ecological, evolutionary & environmental sciences

For a reference copy of the document with all sections, see nature.com/documents/nr-reporting-summary-flat.pdf

Life sciences study design

All studies must disclose on these points even when the disclosure is negative.

Sample size	Sample sizes were determined by ensuring that the number of samples was sufficient to detect statistical differences.
Data exclusions	No data were excluded.
Replication	In vitro experiments were repeated at least three times and a representative data was showed as a technical triplicate.
Randomization	Samples or animals were randomly allocated to each group without bias.
Blinding	This is not relevant to this study since no subjective evaluation of data was performed.

Reporting for specific materials, systems and methods

We require information from authors about some types of materials, experimental systems and methods used in many studies. Here, indicate whether each material, system or method listed is relevant to your study. If you are not sure if a list item applies to your research, read the appropriate section before selecting a response.

Materials & experimental systems

n/a	Involved in the study	
☐	☒	Antibodies
☐	☒	Eukaryotic cell lines
☒	☐	Palaeontology and archaeology
☐	☒	Animals and other organisms
☐	☒	Human research participants
☒	☐	Clinical data
☒	☐	Dual use research of concern

Methods

n/a	Involved in the study	
☒	☐	ChIP-seq
☒	☐	Flow cytometry
☒	☐	MRI-based neuroimaging

Antibodies

Antibodies used

Antibody, Supplyer, Catalog#, clone name
(Western Blotting)
H-Ras, Santa Cruz, sc-29, F235
p16, IBL, 11104, 1H4
STING, CST, #13647, D2P2F
cGAS, CST, 15102, D1D3G
lamin B, Santa Cruz, sc-6217, M-20
DP1, abcam, ab11834, n/a
α-tubulin, Sigma-Aldrich, T9026, DM1A
RNaseH2A, PROTEINTECH, 16132-1-AP, n/a
E2F3, Santa Cruz, sc-878, C-18
mini-AID-tag, MBL, M214-3, 1E4
β-actin, Santa Cruz, sc-47778, C4
(ChIP-qPCR)
Rabbit IgG, CST, #2729, n/a
E2F1, Santa Cruz, sc-193, C-20
E2F3, Santa Cruz, sc-878, C-18
(Immunofluorescence)
Phospho-histone H2A.X (Ser139), Millipore, 05-636, JBW301
phospho-(Ser/Thr) ATM/ATR substrate, CST, #2851, n/a
Lamin B1, abcam, ab16048, n/a
dsDNA, Santa Cruz, sc-58749, HYB331-01
p21 WAF1/CIP1, BD Biosciences, 556430, SX-118
RNaseH2A, OriGene, TA306706, n/a

Validation	Validation data is available at the manufactocterer's websites with published reference and application specific data provided. No new antibodies were used in this study.

Eukaryotic cell lines

Policy information about cell lines

Cell line source(s)	TIG-3 cells, IMR90 cells, Hs68 cells and HEK-293T cells were obtained from the Japanese Cancer Research Resources Bank. BJ and SK-OV-3 cells were obtained from the ATCC. HCT116 cells expressing OsTIR1 and AID-RNaseH2A was provided by Prof. M Kanemaki from National Institute of Genetics, Shizuoka, Japan.
Authentication	Cell lines used in this study were not authentificated.
Mycoplasma contamination	We confirmed the absence of mycoplasma contamination in the cultured cells.
Commonly misidentified lines (See ICLAC register)	No Commonly misidentified cell lines were used in this study.

Animals and other organisms

Policy information about studies involving animals; ARRIVE guidelines recommended for reporting animal research

Laboratory animals	Male C57BL/6J mice were used.
Wild animals	This study did not involve wild animals.
Field-collected samples	No field collected samples were used in this study.
Ethics oversight	All animal procedures were performed using protocols approved by the Japanese Foundation for Cancer Research (JFCR) Animal Care and Use Committee in accordance with the relevant guidelines and regulations (approval number: 1804-05).

Note that full information on the approval of the study protocol must also be provided in the manuscript.

Human research participants

Policy information about studies involving human research participants

Population characteristics	NF1 cells, NF8-2 cells, WF1A cells, WF5 cells and WF8L cells are human skin fibroblasts. NF1 cells from 42-year-old Japanese healthy male and NF8-2 cells from a 45-year-old Japanese healthy male were collected. WF1A cells from a 47-year-old Japanese male who was diagnosed with WS, WF5 cells from a 43-year-old Japanese male who was diagnosed with WS and WF8L cells from a 43-year-old Japanese male who was diagnosed with WS were collected. WF1A, WF5 and WF8L cells contained a homozygous mutation in the WRN gene (Mut4 mutation: c.3139-1G>C).
Recruitment	These cells were collected from patients who provided informed consent for genetic and cell biological analyses.
Ethics oversight	All methods were performed in accordance with the protocols approved by the Institutional Review Board (approval number: 2019-1211) of the JFCR.

Note that full information on the approval of the study protocol must also be provided in the manuscript.
